# Effect of Size on Phase Mixing Patterns in Rapidly Solidified Au–Ge Nanoparticles

**DOI:** 10.3390/nano15120924

**Published:** 2025-06-14

**Authors:** Olha Khshanovska, Vladyslav Ovsynskyi, Aleksandr Kryshtal

**Affiliations:** Faculty of Metals Engineering and Industrial Computer Science, AGH University of Krakow, 30-059 Krakow, Poland; khshanov@agh.edu.pl (O.K.);

**Keywords:** hcp Au, solidification, TEM, alloy nanoparticles, mixing patterns

## Abstract

We investigated the morphological patterns, crystalline structures and their thermal stability in solidified Au–Ge nanoparticles ranging in size from 10 to 500 nm. Liquid Au–Ge alloy nanoparticles with hypoeutectic composition were rapidly cooled from a temperature of 500 °C in a TEM and characterized using advanced TEM techniques. We demonstrated that Au–Ge nanoparticles 10–80 nm in size predominantly solidified into a Janus-like morphology with nearly pure single-crystalline hcp Au and diamond cubic Ge domains. These particles remained stable up to the eutectic temperature, indicating that Ge doping and particle size play key roles in stabilizing the hcp Au phase. In turn, larger nanoparticles exhibited a metastable core–shell morphology with polycrystalline Ge shell and hcp Au-Ge alloy core under solidification. It was shown that the mentioned morphology and crystalline structure evolved into the equilibrium Janus morphology with fcc Au and diamond Ge domains at temperatures above ≈160 °C.

## 1. Introduction

Multiphase alloy nanoparticles incorporate different chemical elements and phases within a single structure, enabling the combination of multiple material properties or the emergence of entirely new ones. These hybrid materials are actively investigated for applications in energy storage [1], energy production [2], catalysis [3], and biomedicine [4]. The chemical and phase mixing patterns in alloy nanoparticles can vary significantly depending on factors, such as particle size, overall composition, thermal history, and synthesis method [5]. Common phase-mixing patterns in binary alloy nanoparticles include core–shell structures, ordered and random alloys, and Janus-like morphologies [6]. These configurations can represent either equilibrium or metastable states.

For instance, Ag–Cu nanoparticles exhibit size-dependent phase-separated morphologies [7,8,9]. Janus morphologies were consistently observed in Ag–Cu nanoparticles ranging from 8 to 20 nm in size [7,8,9]. These smaller particles typically began as single-phase alloys or core–shell-like structures and transformed into Janus configurations upon heating, particularly between 200 °C and 600 °C. In contrast, larger particles (40 nm) formed core–shell structures with Ag-rich shells at intermediate temperatures (300 °C) before eventually transitioning to Janus morphologies at 600 °C [7]. In Au–Sn systems above 900 °C, nanoparticle morphology and phase fractions were influenced by particle size, where smaller nanoparticles (20 nm) exhibited single-phase morphology, while larger nanoparticles (50 nm) exhibited Janus structures [10]. In the Cu–Ni system, calculations show that nanoparticles with a size of 4 nm exhibit a mixed morphology, while those with a size of 10 nm adopt a Janus morphology at a synthesis temperature of 523 °C [11].

The Au–Ge system, in particular, has attracted considerable attention due to its unique phase diagram, which includes a size-dependent miscibility gap and a low eutectic temperature (~361 °C) [12]. In nanoscale systems, a Janus-like structure is typically observed in Au–Ge nanoparticles up to 25 nm in size. The Au-rich domains have been reported to be compositionally inhomogeneous and consist largely of a hexagonal Au–Ge alloy, likely near the eutectic composition (28 at.% Ge) [13]. The Janus morphology is considered the equilibrium structure for small Au–Ge nanoparticles. However, a study [1] showed that rapid cooling can result in a metastable, compositionally mixed Au–Ge structure. Remarkably, this mixed state can revert to the equilibrium bilobed Janus structure upon thermal annealing at 300 °C for just 10 s in 9 nm particles. It was concluded that phase transformations in the Au–Ge system are reversible and strongly dependent on the thermal history. In situ TEM experiments showed that 60 nm Au particles catalyze Ge growth at temperatures as low as 150 °C, resulting in asymmetric, bilobed structures [14]. The formation of equilibrium Janus-like Au–Ge structures (~116 nm in size) has been reported during deposition of Ge onto Au islands on Si(100) substrate [15]. It was shown that the formation of an Au–Si nanoalloy is necessary for the development of phase-separated Au–Ge bilobed nanostructures.

The hcp phase of Au, while rare, has been observed in several studies involving Au–Ge nanoparticles [16,17], especially at the tips of Ge nanowires [18,19,20,21]. It has been shown that the composition of the 40–50 nm particles that adopt the hcp structure at the tips of Ge nanowires is almost pure Au, containing less than 4 at.% Ge. [18,19,21]. Pure hcp Au phase segments with well-defined crystallographic orientation and grain size ranging from 50 nm to 110 nm were reported in Au nanoparticles formed in the process of thermally induced self-assembly of a thin Au layer on Ge(001) monocrystal [16]. A phase transition from fcc to hcp of structure was theoretically predicted for Au at a pressure of 241 GPa [22].

Recently, we studied the solidification pathway of individual Au–Ge nanoparticles using in situ TEM techniques [17]. It was revealed that the solidification of undercooled nanoparticles occurred over a temperature range, rather than at a single temperature, and proceeded through an intermediate hcp Au-based phase. This observation opens up possibilities for tuning the phase and morphological structures in Au–Ge nanoparticles, potentially enabling highly unusual combinations of phase structures and chemical mixing patterns. Therefore, the aim of this work was to investigate the effect of particle size on phase mixing patterns and their thermal stability in solidified Au–Ge nanoparticles.

## 2. Materials and Methods

A sample containing Au–Ge nanoparticles of various compositions and sizes was used in this study. It was prepared as follows. First, a thin Au–Ge film was formed by sequential deposition of components in a vacuum of 1 × 10^−6^ Torr using a DC magnetron sputtering system (Prims 032, PREVAC, Rogów, Poland). The total film thickness was ≈4.75 nm, with an overall composition of ≈22.5 at.% Ge as measured using a quartz crystal microbalance. The deposition was carried out onto a room-temperature silicon nitride (Si_3_N_4_) substrate of a nanochip (DENSsolutions WildfireHB GT, Delft, The Netherlands). After deposition, the sample was transferred to a transmission electron microscope (TEM). The nanoparticles were formed by melting the Au–Ge film inside the TEM by heating the sample to 420 °C. This caused dewetting of the thin film and the formation of well-separated liquid Au–Ge nanoparticles, ranging in size from a few nanometers to approximately 500 nm. The composition of nanoparticles smaller than ~80 nm spanned from hypo- to hyper-eutectic, whereas the larger nanoparticles exhibited a hypo-eutectic composition.

The morphology, crystalline structure, and composition of nanoparticles were characterized over a temperature range of 20–500 °C using a probe Cs-corrected Titan Cubed G2 60-300 (Thermo Fisher Scientific, Waltham, MA, USA) electron microscope operated at 300 kV. The microscope was fitted with a MEMS-based heating holder (Wildfire D6, DENSsolutions, Delft, The Netherlands) for in situ experiments. The Au–Ge particles were solidified by rapid cooling from 500 °C directly to room temperature. The cooling rate reached 200 °C/ms, according to the holder specification. In some cases, a gradual cooling at a rate of 1 °C/s was used. High-angle annular dark-field (HAADF) detector with a collection angle of 50–200 mrad was used for imaging in STEM. The overall composition of the nanoparticles was measured in the liquid state using EDX analysis. Instead of point analysis, EDX maps with a resolution of 64 × 64 pixels were acquired on the central area of the Au-Ge NPs to minimize radiation damage. Each map was collected over 120 s at a beam current of 0.152 nA. EDX mapping was used for studying distribution of chemical elements in solidified nanoparticles. The maps were acquired with a beam current of 0.1 nA. For nanoparticles smaller than 80 nm, an acquisition time of 30 min was used, resulting in a total radiation dose of 2.3 × 10^8^ e⁻/nm^2^ per map. For larger particles, the acquisition time was reduced to 10 min, corresponding to a total electron dose of 4.0 × 10^6^ e⁻/nm^2^. All EDX datasets were processed using a Python-based script developed with the open-source HyperSpy library (version 2.0.2) running on Python 3.12.9 [23].

The content of Ge in Au domains of Janus nanoparticles was measured using EELS. A core-loss spectrum was acquired with an energy dispersion of 1 eV/channel using a convergent and collection semi-angles of 13 and 30 mrad, respectively. The spectrum was obtained by summing 20 individual frames, each recorded with a 2 s acquisition time, resulting in a total integration time of 40 s. The beam current during EELS acquisition was approximately 250 pA. After subtraction of the background and removing multiple inelastic scattering (Fourier-ratio deconvolution method), a ratio of atomic concentrations was calculated using the Gatan Digital Micrograph analysis software package (version 3.23.1521.0).

Structural characterization was performed via selected area electron diffraction (SAED) and high-resolution HAADF-STEM imaging followed by Fourier transformation (FT) analysis. The diffraction patterns were indexed with the help of Java Electron Microscopy Software (JEMS, version 5.1931u2024b22) [24].

## 3. Results

### 3.1. Solidification Patterns in 10–80 nm Au–Ge Nanoparticles

**Figure 1:**
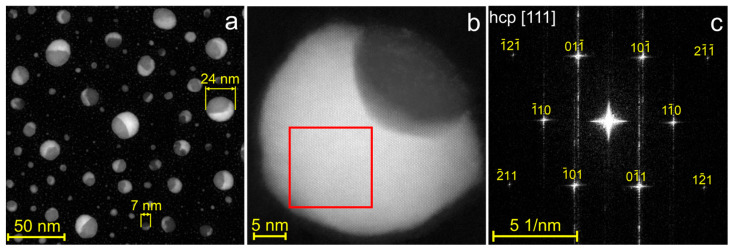
HAADF-STEM images of Au–Ge nanoparticles: (**a**) a collection of nanoparticles with some particle sizes labeled, and (**b**) a single nanoparticle exhibiting the typical morphology for this size range. (**c**) Fourier transform (FT) of the region marked by the red square in (**b**), indexed to hcp lattice along the [111] zone axis.

Figure 1a shows an overview HAADF-STEM image of solidified Au–Ge nanoparticles with sizes below ≈80 nm on a Si_3_N_4_ substrate. The image contrast revealed a Janus-like morphology—characterized by partial or complete demixing of the components and the formation of distinct phases within individual nanoparticles—which dominated in this size range. Despite the as-deposited film having a fixed composition of approximately 22.5 at.% Ge, the composition of individual nanoparticles varies widely, as evidenced in Figure 1a, where the Ge-to-Au ratio differs across the particle population. Consequently, nanoparticles with both hypo-eutectic and hyper-eutectic compositions are present in Figure 1a. Nevertheless, the Janus-like morphology remains consistent regardless of composition. Notably, the formation of the Janus morphology was independent of the cooling rate, occurring under both quenching and gradual cooling conditions.

Figure 1b shows a representative Au–Ge nanoparticle at higher magnification. The nanoparticle had a diameter of 55 nm and contained 24 at.% Ge. Upon solidification, a distinct biphasic morphology with an incoherent phase boundary emerged. FT analysis of the high-resolution image revealed a hexagonal phase in the [111] orientation for the Au domain, with lattice constants a = 0.29 nm and c = 0.49 nm (Figure 1c). The measured values had an estimated uncertainty of approximately 5%. We analyzed nearly a dozen Au–Ge nanoparticles with Janus-like morphologies and sizes ranging from 10 to 80 nm. All exhibited a single-crystalline hcp structure in the Au domains and a diamond cubic structure in the Ge domains.

The composition of phases in Janus-like Au–Ge nanoparticles was examined using EDX and EELS techniques. Representative EDX spectra from the Au and Ge domains of a Janus nanoparticle are shown in the Supplementary Information (Figure S1). Quantitative analysis of the spectra revealed that the Au–Ge heterodimer is composed of Au and Ge primary solid solutions. Specifically, the Ge content in the hcp Au domain varied between 2–6 at.% across different particles. Conversely, the Au content in the Ge domain did not exceed 3 at.%. It is worth noting that the accuracy of EDX analysis for elements present at low concentrations in nanoparticles may be as poor as 50% [25]. Additionally, parasitic X-ray signals from adjacent regions of the Janus nanoparticle can affect EDX quantification results. To verify the content of Ge in hcp Au, core-loss EELS spectra were acquired from Au domains of several Janus nanoparticles with sizes 50–75 nm. A representative EELS spectrum, showing Ge-L and Au-M edges, is provided in the Supplementary Information (Figure S2). Quantification of spectra gave Ge concentration of 3–4 at.% in the hcp Au phase, which is consistent with the EDX data.

**Figure 2:**
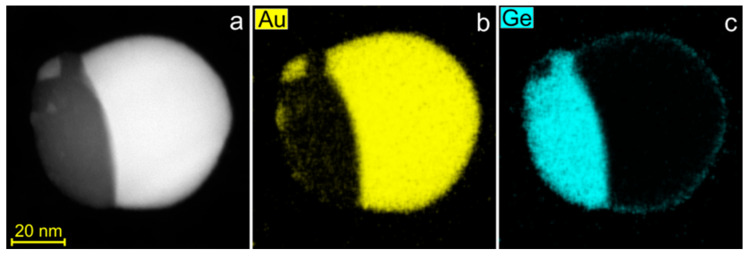
HAADF-STEM image (**a**) and EDX elemental maps (**b**,**c**) for solidified Au–Ge nanoparticle.

EDX mapping was used to examine the compositional homogeneity of phases in Janus nanoparticles. Figure 2a presents a HAADF-STEM image of a 75 nm Au–Ge nanoparticle with an overall composition of 23.6 at.% Ge. The corresponding EDX maps for Au and Ge are shown in Figure 2b,c. These maps reveal phase separation into a compositionally homogeneous Au domain with a Ge content of ≈2.5 at.%, while the Ge domain exhibits the presence of Au precipitates at the surface. The mean Au content in the central area of the Ge domain was about 1 at.%.

**Figure 3:**
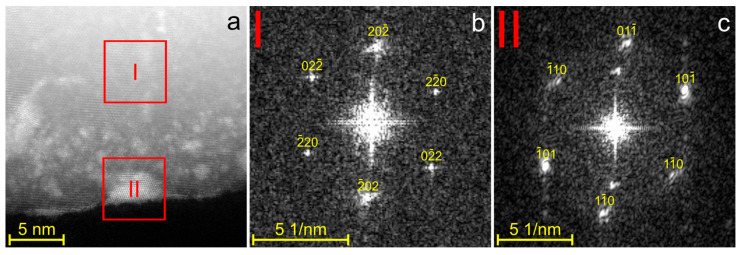
(**a**) High-resolution HAADF-STEM image of the Ge-rich region of the Au–Ge Janus-like nanoparticle, showing gold precipitates. (**b**,**c**) Fourier transforms from regions I and II. The FT pattern in (**b**) is indexed to the Ge diamond cubic lattice along the [111] direction, while the FT pattern in (**c**) is indexed to the hcp Au–Ge structure along the [111] direction.

The Ge domain in Janus nanoparticles was further examined using high-resolution HAADF-STEM imaging (Figure 3a). Owing to the large difference in atomic numbers between Au and Ge, the gold precipitates are clearly visible in Figure 3a. These precipitates are located primarily on the surface of the Ge region (Figure 2a and Figure 3a). The Fourier transform (FT) pattern of region II in Figure 3a is shown in Figure 3c and reveals the hcp structure of the Au-based precipitate. The FT from region I, corresponding to the Ge domain, is shown in Figure 3b and displays the equilibrium diamond cubic phase of Ge.

**Figure 4:**
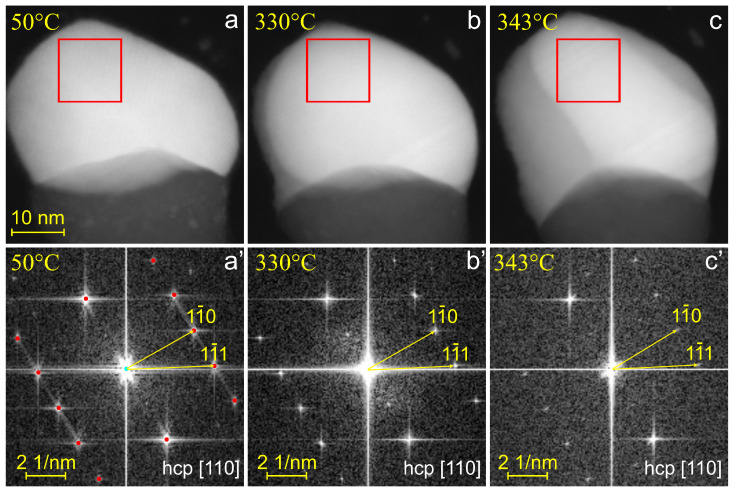
HAADF-STEM images (**a**–**c**) and corresponding FT patterns (**a’**–**c’**) of the Au–Ge particle at different temperatures during the heating process. The FT patterns were obtained from the region marked by red squares in the HAADF images. In (**a’**), red dots indicate the simulated diffraction pattern of the hcp [110] structure. The scale bar applies to all HAADF-STEM images in the top row.

The thermal stability of the hcp Au structure was studied using in situ TEM heating over a temperature range of 20 to 360 °C. Figure 4 shows selected high-resolution HAADF-STEM images of the Au domain within the same Janus nanoparticle at different temperatures. At 50 °C, the Au exhibited an hcp structure in the [110] orientation. Upon heating, eutectic melting initiated at the triple junction at 330 °C, as evidenced by the appearance of a liquid phase in Figure 4b. Despite this, the crystalline structure of the Au domain remained unchanged, as confirmed by Fourier transform (FT) analysis, which continued to reveal the hcp lattice (Figure 4b’). As the temperature increased further, the liquid phase progressively expanded, and the Au domain decreased in size, yet it maintained its crystalline structure (Figure 4c,c’). Therefore, we conclude that the hcp Au structure remained stable up to the eutectic temperature in 10–80 nm size nanoparticles.

### 3.2. Solidification Patterns in Au–Ge Nanoparticles with Sizes from 80 to 500 nm

**Figure 5:**
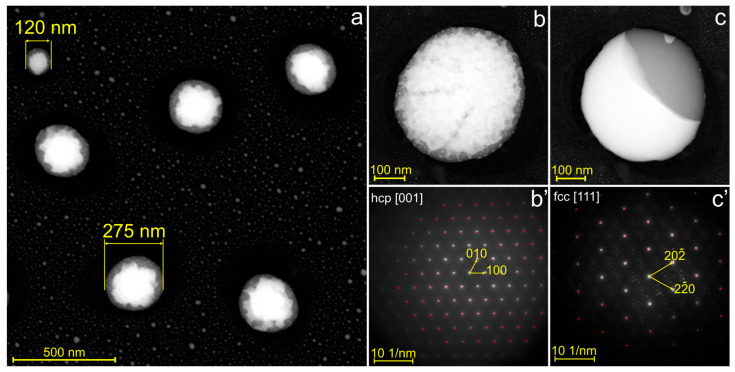
(**a**) HAADF-STEM image of Au–Ge core–shell nanoparticles. Labels indicate the smallest (~120 nm) and largest (~275 nm) particles within the field of view. (**b**,**c**) HAADF-STEM images of a representative 400 nm Au–Ge nanoparticle: as solidified (**b**) and after annealing at 350 °C (**c**). (**b’**,**c’**) Corresponding SAED patterns of the particle, indexed to the hcp [001] and fcc [111] zone axes, respectively.

**Figure 6:**
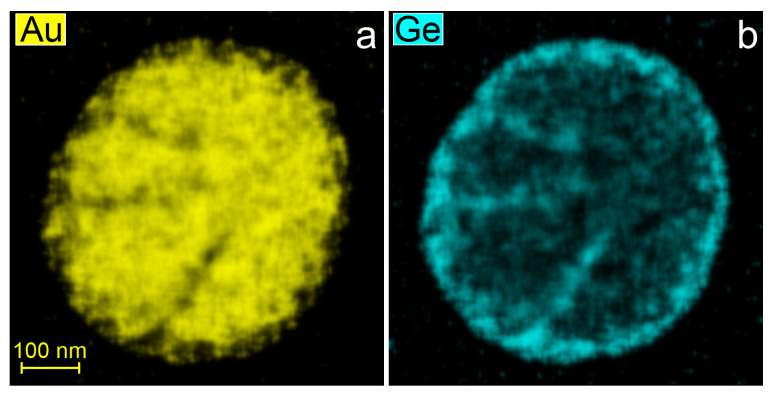
EDX elemental maps (**a**,**b**) of the Au–Ge nanoparticle shown in Figure 5b.

Separation of phases during rapid solidification of Au–Ge nanoparticles ranging in size from 80 to 500 nm resulted in the formation of a core–shell-like morphology, with a Ge-rich shell and an Au-rich core. An overview HAADF-STEM image of nanoparticles with sizes ranging from ~120 to ~275 nm is shown in Figure 5a. A single Au–Ge particle is shown in Figure 5b at higher magnification. It has a size of approximately 400 nm and a hypo-eutectic composition of 23 at.% Ge. Additional images demonstrating the predominant core–shell solidification morphology in hypo-eutectic Au–Ge nanoparticles are available in the Supplementary Information (Figures S3 and S4). Meanwhile, subtle contrast variations in the HAADF-STEM images suggest that the particle cores are compositionally non-uniform. Electron diffraction analysis showed that the Ge-rich shell had a polycrystalline diamond structure (Figure S3b in the Supplementary Information), while the Au-rich core exhibited a single-crystalline hcp phase, as revealed by Figure 5b′ and Figures S3b and S4b in the Supplementary Information. The lattice constants measured from SAED patterns were a = 0.286 nm for the hcp structure, and a = 0.565 nm for the diamond cubic structure. The core–shell morphology is further confirmed by EDX mapping of chemical elements in the nanoparticle shown in Figure 5b. The Ge-rich shell and Au-rich core are visible in Figure 6a,b. The EDX maps also revealed that complete phase separation did not occur: the Au content in the Ge shell varied between 8 and 40 at.%, while the core consisted primarily of gold, with the Ge content ranging from 10 to 20 at.% across the core. Hence, we concluded that the core of the nanoparticles consists of an Au–Ge hcp alloy.

The core–shell morphology and Au–Ge alloy were not stable for particles in this size range. Upon heating the particle shown in Figure 5b to a pre-melting temperature, it transformed into a Janus-like morphology, featuring single-crystalline fcc Au with a lattice constant a = 0.404 nm and diamond-structured Ge on opposite sides. This transformation is evident in Figure 5c,c′, which show the HAADF image and corresponding diffraction pattern of the particle annealed at 350 °C. EDX analysis of the fcc Au domain revealed a Ge content of approximately 1.5 at.%. Therefore, annealing to a pre-melting temperature followed by slow cooling provides a means to achieve the equilibrium phase structure and elemental distribution in Au–Ge nanoparticles with sizes between 80 and 500 nm (Figure 5c,c′).

**Figure 7:**
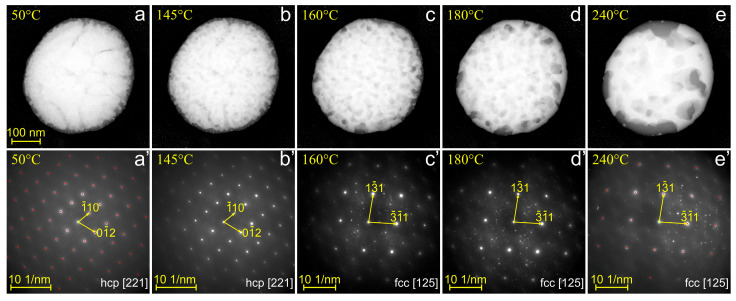
HAADF-STEM images (**a**–**e**) and corresponding SAED patterns (**a’**–**e’**) of the same Au–Ge nanoparticle at different temperatures during the heating process. The temperature is indicated in the upper-left corner of each image. In (**a’**), red dots indicate the simulated diffraction pattern of the hcp [221] structure. The scale bar is identical for all images (**a**–**e**).

To gain insight into the thermal decomposition of a metastable Au–Ge alloy, we performed an additional thermal-cycling experiment on the nanoparticle shown in Figure 5. First, the particle was melted and then rapidly cooled from 500 °C. Subsequently, it was gradually heated to 240 °C, and its morphology and crystal structure were recorded at intervals of 15–20 °C. Selected images and diffraction patterns are presented in Figure 7.

At 50 °C, the nanoparticle exhibits a core–shell morphology (Figure 7a), similar to that shown in Figure 5a. This observation demonstrates the reproducibility of particle morphology across different thermal cycles. The SAED pattern confirms the hcp Au–Ge phase, viewed along the [221] zone axis (Figure 7b’). As the temperature increases to 145 °C, the fine structure of the particle begins to coarsen, as indicated by the variation in image contrast between Figure 7a and b. Notably, the hcp structure of the particle remains stable up to this temperature.

At higher temperatures, the morphological evolution was accompanied by structural transformations. Specifically, a transformation from the hcp structure to face-centered cubic (fcc) structure is evident in Figure 7c’, which shows fcc Au viewed along the [125] zone axis. Please note the emergence of additional reflexes in Figure 7c’, likely originating from Ge domains. Further increases in temperature did not alter the crystalline structure of the particle (Figure 7d’,e’) but did accelerate the morphological transformation (Figure 7d,e). At 240 °C (Figure 7e), the particle exhibits a mixture of Au- and Ge-based domains, indicating incomplete phase separation. With continued heating, the particle evolves into the Janus morphology shown in Figure 5b.

Similar measurements were performed on several Au–Ge nanoparticles of hypo-eutectic composition. In each case, the hcp-to-fcc phase transformation was observed, although the transition temperature varied from particle to particle within the range of 140–180 °C. These observations indicate that the hexagonal Au–Ge alloy remains stable up to approximately 160 ± 20 °C in nanoparticles with sizes ranging from 80 to 500 nm.

## 4. Discussion

Phase separation during the solidification of Au–Ge nanoparticles is influenced by several interconnected factors, including particle size, cooling rate, solidification temperature, initial composition, diffusion rate, and interfacial energies. Therefore, we aimed to minimize the number of factors influencing the morphology of solidified nanoparticles. The nanoparticles were cooled from a temperature of 500 °C, well above the eutectic temperature of 361 °C, ensuring a single-phase liquid state across a broad range of alloy compositions (23–49 at.% Ge). Solidification of liquid Au–Ge nanoparticles occurs at large supercooling, typically around 200 °C [17]. The high cooling rates used in this study limited the time available for diffusional growth of the separating phases, favoring the ‘freezing’ of the solidification morphology. Under these conditions, nanoparticle size becomes one of the main factors affecting separation of phases during solidification.

The formation of Janus morphology in nanometer-sized Au–Ge particles was an expected observation, although the upper size limit of 80 nm is somewhat higher than typically reported in the literature of about 25 nm [1,13]. Au and Ge are nearly insoluble in the solid state [12], and the volume diffusion coefficient in nanomaterials exceeds the bulk value by several orders of magnitude [26], enabling the diffusion-induced phase separation even at room temperature. In this case, the interfacial energies dictate the equilibrium structure in strongly segregating binary alloys [27]. Janus-like morphologies were observed in colloidal Au–Ge nanoparticles [1], as well as in nanoparticles embedded in silica [13]. However, the presence of highly stable single-crystalline hcp Au-based domains in Janus nanoparticles is a new observation. Recently, we observed hcp Au nuclei in undercooled liquid Au–Ge nanoparticles with sizes around 160 nm. Thermodynamic analysis indicated that hcp Au in liquid nanoparticles may be stabilized by size and atomic packing factors [17,28,29]. At the same time, pure Au nanoparticles exhibit their native fcc structure across the entire range of sizes and temperatures [30]. Thus, the size factor alone is unlikely to stabilize the hcp Au structure in Janus Au–Ge nanoparticles.

We speculate that Ge doping of Au, combined with the small particle size, helps stabilize the hcp crystal structure in Au–Ge nanoparticles. Indeed, a minor (2–6 at.%) content of Ge was consistently found in the Au domains of the Janus nanoparticles. This concentration is too low to form an Au–Ge hcp alloy, which typically requires a Ge content in the range of 16–25 at.% [17]. Therefore, the Au domains in the Janus nanoparticles could be considered a primary solid solution of hcp Au. Although the equilibrium solubility of Ge in fcc Au does not exceed 0.3 at.% at room temperature [12], the solubility of Ge in hcp Au is, to the best of our knowledge, unknown. However, alloy nanoparticles and films often exhibit enhanced mutual solubility due to size reduction [9,17]. The Ge dopants alter the electron density, which in turn can favor a different Au crystal packing in nanoscale particles. Furthermore, the significant atomic size difference between Au and Ge induces local strain, potentially shifting the energy balance between competing fcc and hcp phases. More detailed studies of dilute Au–Ge solid solutions in nanoparticles are needed to gain further insight into the phenomenon.

The rapid solidification of larger nanoparticles (approximately 80–500 nm in size) results in non-equilibrium quasi core-shell morphology with polycrystalline Ge-rich shell and single crystalline hcp Au–Ge alloy core, i.e., the complete phase separation did not occur. At the solidification temperature of approximately 200 °C, volume diffusion in solid nanoparticles of this size range is limited. Consequently, diffusional phase separation occurs primarily in the surface and near-surface regions, forming a Ge-based shell (Figure S2 in the Supplementary Information). Phase separation within the nanoparticle volume likely proceeds via spinodal decomposition, which begins spontaneously and uniformly throughout the material by composition fluctuations. This is supported by the characteristic contrast in the HAADF-STEM image in Figure 5a as well as by EDX maps of the core in Figure 6, which demonstrate the initial stage of the phase separation in rapidly solidified nanoparticles. Naturally, this morphology is metastable, and it gradually transforms into the equilibrium Janus morphology (Figure 5b) as volume diffusion is activated upon heating.

Another key result of this work is the determination of the terminal thermal stability of the hcp Au–Ge alloy in core–shell nanoparticles, which was found to be ≈150 °C for the particle shown in Figure 7. This temperature is substantially higher than values reported in the literature. For instance, decomposition of the Au–Ge phase in a silica matrix has been observed to begin at 80 °C [1]. Notably, in our nanoparticles, coarsening of the compositional fluctuations into larger domains began before the temperature of 150 °C (Figure 7b), indicating that the morphological transformation precedes the structural transition from hcp to fcc. Thus, the growth of Au-rich domains along with precipitation of Ge is required to induce the decomposition of the hcp Au–Ge alloy into the equilibrium fcc Au and Ge phases.

## 5. Conclusions

This study examined the morphological and structural evolution of Au–Ge nanoparticles ranging in size from 10 to 500 nm during rapid solidification from the liquid state. We found that smaller nanoparticles (10–80 nm) solidified into a Janus-like morphology comprising almost pure single-crystalline hcp Au and diamond cubic Ge domains, regardless of the cooling rate. The high-temperature stability of the hcp Au phase was attributed to Ge doping and small particle size; that is, a few atomic percent of Ge stabilized the hcp Au structure in nanoparticles. In contrast, rapid solidification of larger nanoparticles resulted in a metastable core–shell morphology, with a polycrystalline Ge shell and an hcp Au–Ge core. The latter decomposed above approximately 160 °C, and the nanoparticles adopted their equilibrium heterodimer shape, featuring fcc Au and diamond cubic Ge domains. These findings expand our understanding of solidification morphologies and enable the tuning of phase and morphological structures in Au–Ge nanoparticles, allowing for the realization of highly unusual combinations of phase structures and chemical mixing patterns.

## Data Availability

Dataset is available on request from the authors.

## Supplementary Materials

The following supporting information can be downloaded at: https://www.mdpi.com/article/10.3390/nano15120924/s1, Figure S1. EDX spectra acquired from the Au-rich (black) and Ge-rich (red) domains of a Janus Au–Ge nanoparticle with a size of 75 nm on a Si_3_N_4_ substrate. Figure S2. Core-loss EELS spectrum acquired from the hcp Au domain of 80 nm Janus Au–Ge nanoparticle on a Si_3_N_4_ substrate at room temperature. Figure S3. HAADF-STEM image (a) and SAED pattern (b) of the Au–Ge particle with core–shell morphology and an overall composition of 27 at.% Ge. Figure S4. HAADF-STEM image (a), SAED pattern (b), and EDX spectrum (c) of the Au–Ge particle with core–shell morphology.
