# Supplementary material for: Effect of Size on Phase Mixing Patterns in Rapidly Solidified Au–Ge Nanoparticles
Source: Nanomaterials (Basel). 2025 Jun 14;15(12):924. doi: 10.3390/nano15120924 (PMC12195838; doi:10.3390/nano15120924)
Supplement: Supplementary file 1 [file nanomaterials-15-00924-s001.zip › nanomaterials-3667150-supplementary.pdf]

Supplementary Information for

Effect of size on phase mixing patterns in rapidly solidified Au–Ge nanoparticles

*Olha Khshanovska, Vladyslav Ovsynskyi, and Aleksandr Kryshtal**

Faculty of Metals Engineering and Industrial Computer Science, AGH University of Krakow, Al. Mickiewicza 30, Krakow 30-059, Poland

*Correspondence: kryshtal@agh.edu.pl

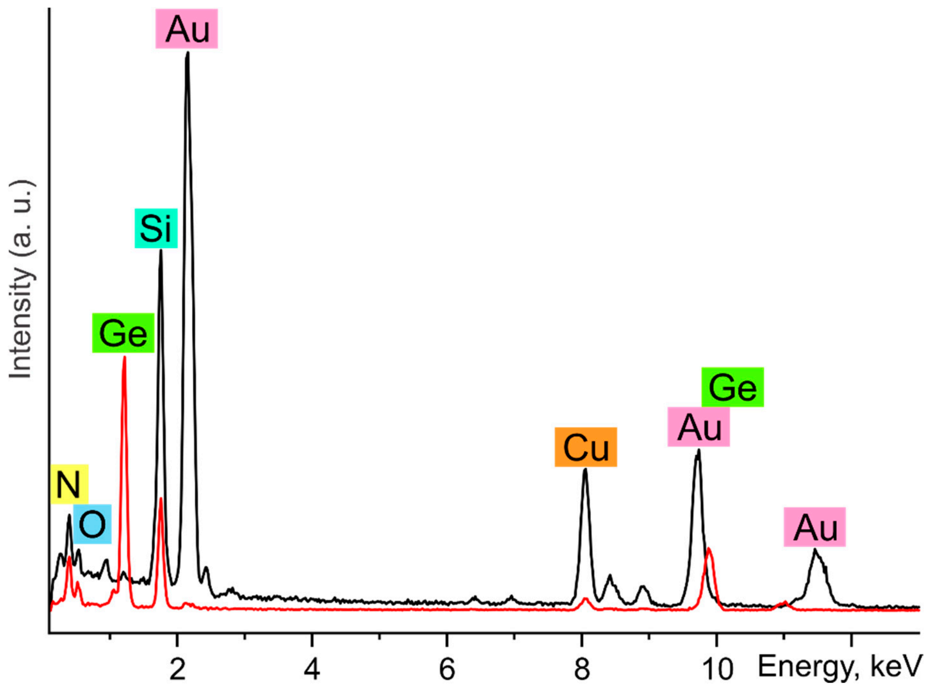

Figure S1. EDX spectra acquired from the Au-rich (black) and Ge-rich (red) domains of a Janus Au–Ge nanoparticle with a size of 75 nm on a Si_3N_4 substrate. The spectra were collected at room temperature. The spurious Cu peaks originate from the *in situ* holder cradle.

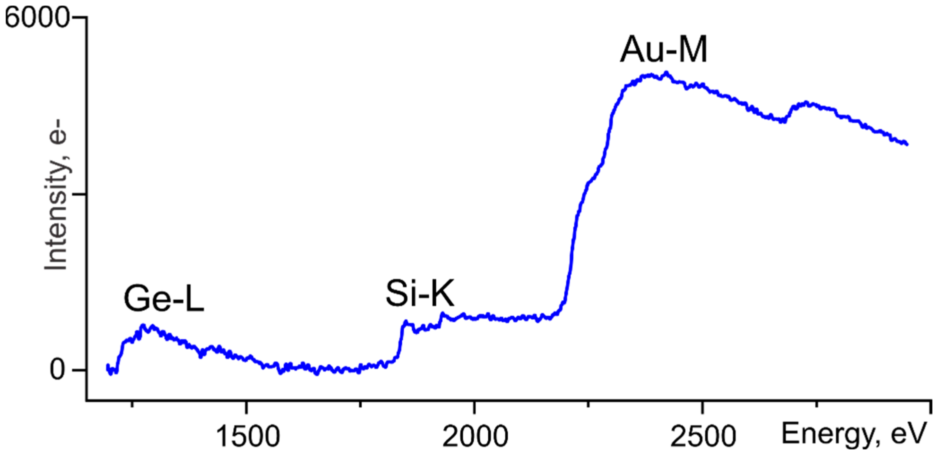

Figure S2. Core-loss EELS spectrum acquired from the hcp Au domain of 80 nm Janus Au–Ge nanoparticle on a Si_3N_4 substrate at room temperature. Quantification of the spectrum yielded approximately 4 at.% Ge and 96 at.% Au in the hcp phase.

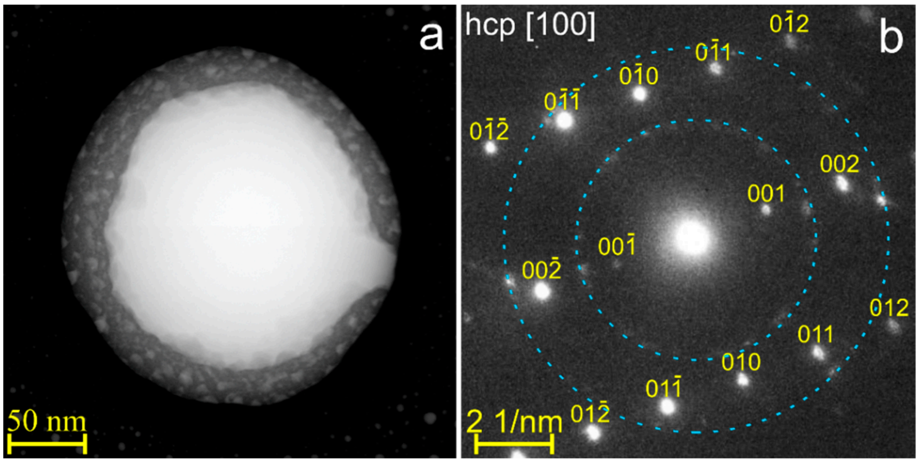

Figure S3. HAADF-STEM image (a) and SAED pattern (b) of the Au–Ge particle with core-shell morphology and an overall composition of 27 at.% Ge. Diffraction pattern is indexed to hcp [100] direction. Dotted rings in (b) correspond to polycrystalline Ge.

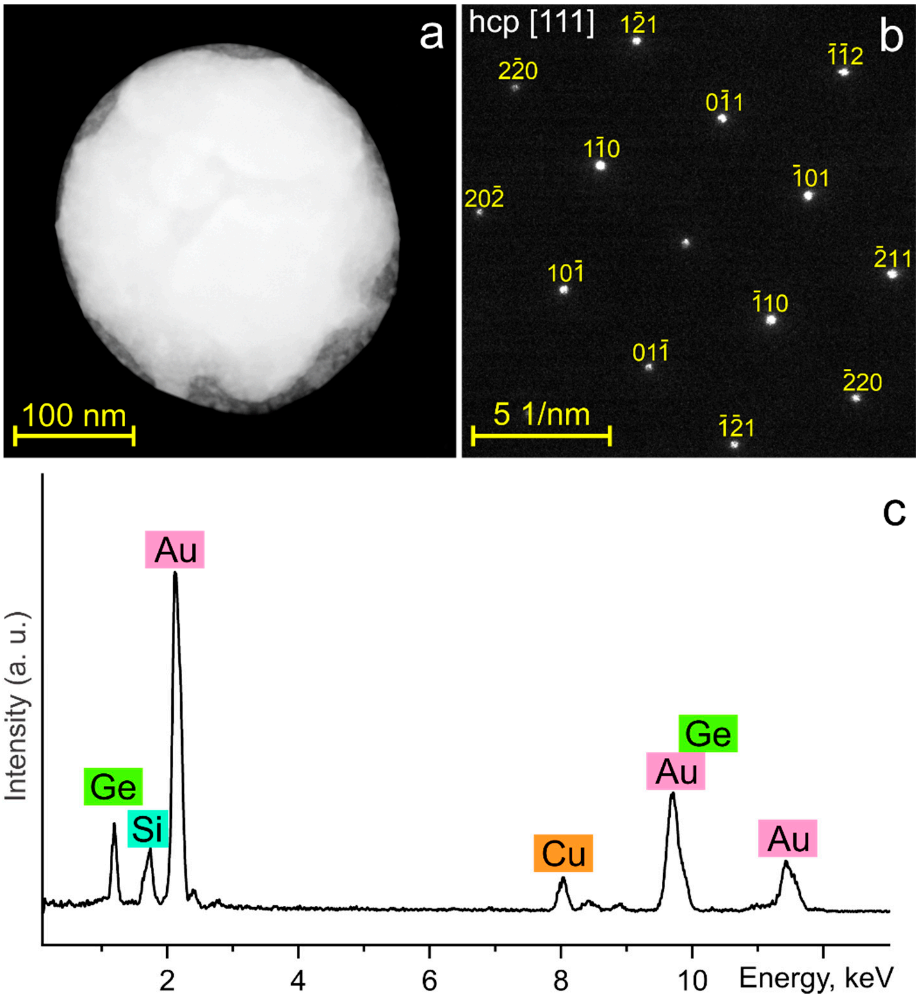

Figure S4. HAADF-STEM image (a), SAED pattern (b), and EDX spectrum (c) of the Au–Ge particle with core-shell morphology. The diffraction pattern is indexed to the hcp [111] zone axis. The EDX spectrum was acquired at 400 °C and corresponds to the liquid state of the particle. Quantitative analysis indicates a composition of 26.6 at.% Ge.
